# Supplementary material for: Helraiser intermediates provide insight into the mechanism of eukaryotic replicative transposition
Source: Nat Commun. 2018 Mar 29;9:1278. doi: 10.1038/s41467-018-03688-w (PMC5876387; doi:10.1038/s41467-018-03688-w)
Supplement: Supplementary file 1 — Supplementary Information(PDF 1172 kb) [file 41467_2018_3688_MOESM1_ESM.pdf]

Helraiser intermediates provide insight into the mechanism of eukaryotic replicative transposition

Grabundzija *et al.*

Supplementary Information

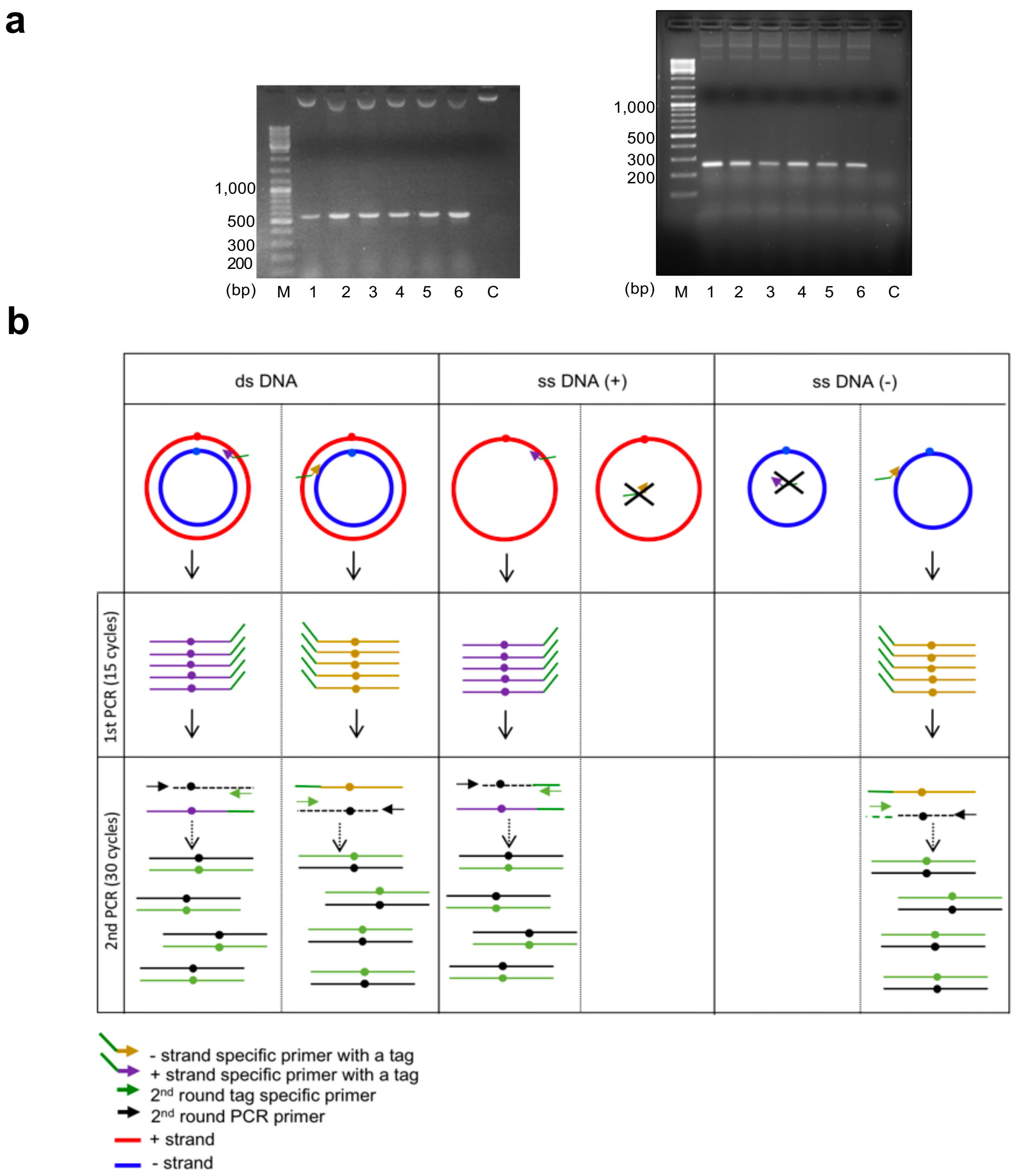

Supplementary Figure 1 PCR detection of transposon donors and circles. (**a**) Uncropped images from the **Fig. 2a** (left) and **b** (right). C, no template control. (**b**) Schematic outline of the strand-specific PCR procedure for detection of transposon-end junctions. In the first asymmetric PCR the LMW sample is divided into two PCR reactions where each reaction is probed with only one tagged primer that is complementary either to the plus (red) or the minus (blue) strand of transposon circles. Primer “tag” sequence is shown as a green line; purple arrow represents the primer complementary to the plus strand; gold arrow represents primer complimentary to the minus strand of transposon circles; purple and gold lines represent products of the first PCR. In the second PCR round, single-stranded products of the first PCR are amplified using second round PCR primers (black arrows) and a tag primer (green arrows). Double-stranded products of the second PCR are shown as green and black lines. Tag primer and the second round primer binding sites encompass transposon end junction position (shown as a full circle), facilitating the detection of *Helraiser* circles. In the case of the double-stranded template, both first round primers are expected to give rise to amplification products in the second PCR round. In contrast, in case of the single-stranded templates, only those reactions probed with the primers complementary to the strand present in the first PCR reaction, would result in amplification in the second round of PCR.

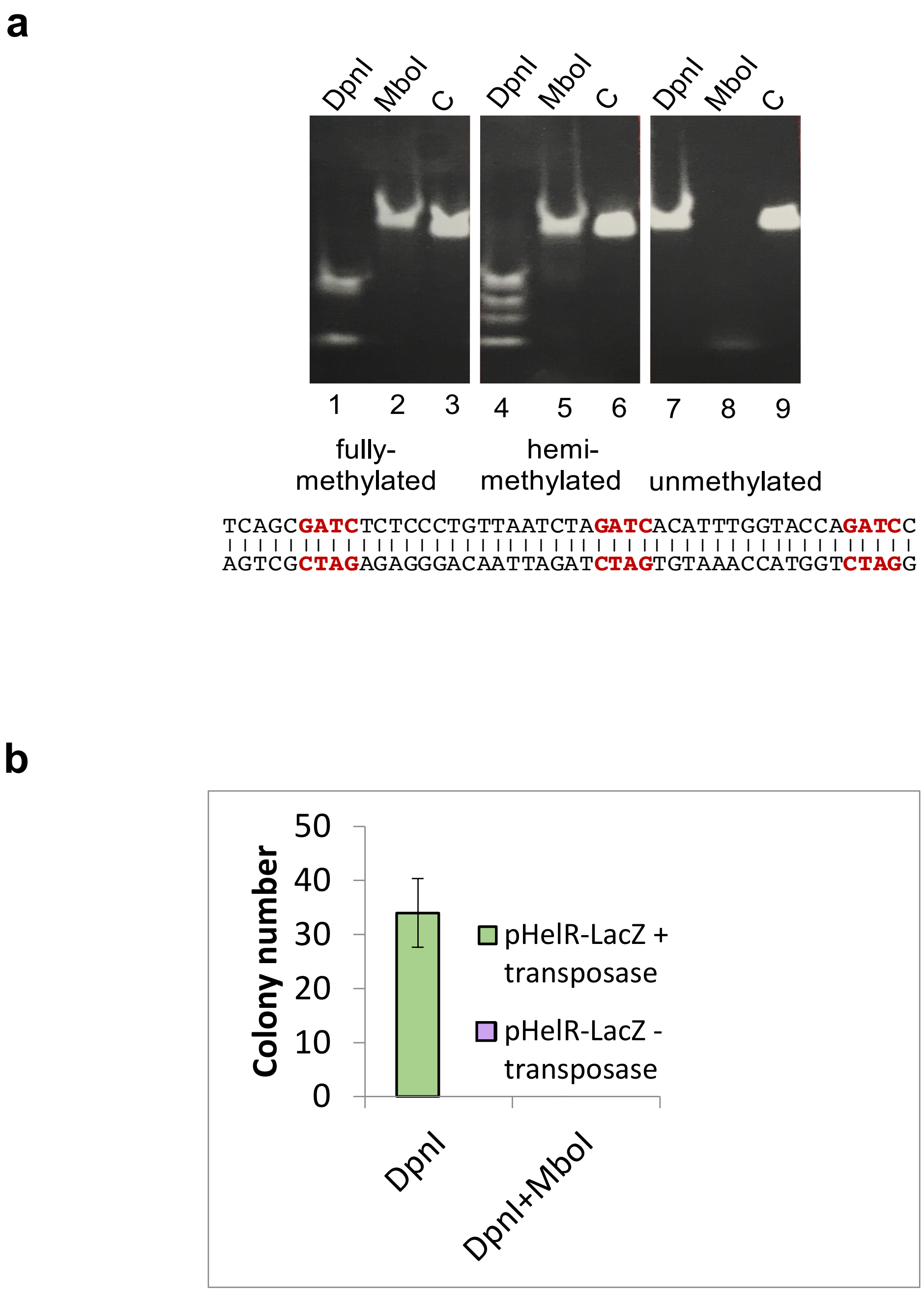

Supplementary Figure 2 *Dpn*I and *Mbo*I digestion of *dam*-methylated DNA (**a**) Control *Dpn*I and *Mbo*I cleavage of a methylated 47 bp oligonucleotide. DNA sequence is as shown with methylated sites indicated in red. (**b**) Results of the *Dpn*I replication assay with pHelR-Cam-LacZ plasmid. Data are presented as a mean± s.e.m., n=3 biological replicates.

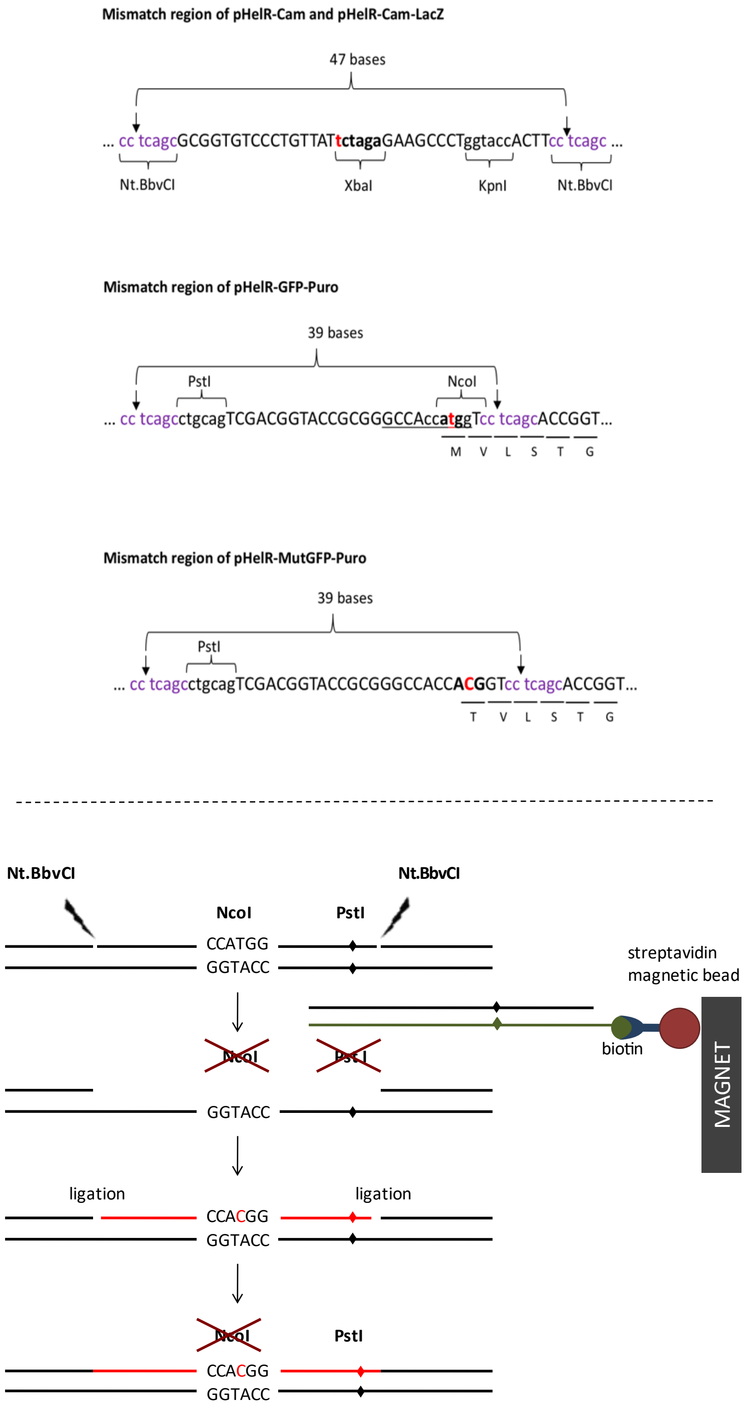

Supplementary Figure 3 *Helraiser* heteroduplex donors. Top: Mismatch regions of the four template plasmids (pHelR-Cam, pHelR-Cam-LacZ, pHelR-GFP-Puro and pHelR-MutGFP-Puro) used for generation of the heteroduplex transposon donors. Exchanged sequence on the plus strand of the template plasmid is shown. The number of exchanged bases is indicated above the sequence. Enzyme recognition sites are in lower case. Nt.BbvCI recognition sites are in purple; black arrows mark Nt.BbvCI nicking sites. Enzyme recognition sites overlapping the mismatch position (in red) are in bold. In the mismatch region of pHelR-GFP-Puro the Kozak sequence is underlined and amino acid sequence of mutated region of the GFP is shown including the new start codon, four inserted amino acids and the original methionine that was changed into glycine. In the mismatch region of pHelR-MutGFP-Puro mutated start codon is shown in bold with the mismatch position indicated in red. **Bottom**: Outline of the heteroduplex generation method is shown using pHelR-GFP-Puro template plasmid. Black lines represent ds DNA strands. Position of the PstI restriction site is indicated by diamond shapes. Oligonucleotide carrying mismatched nucleotide (in red) is shown as a red line. Sequence of the NcoI recognition site overlapping the mismatch position in heteroduplex donor is shown. Biothinylated oligo complementary to the exchanged strand is shown as a green line.

Application	Name	Sequence 5’-3’
PCR	Hel1	cctcctggggcgcttgacacctgcg
	Hel5	tcatctctcacacttctattatagag
	pUC57	ctctgacacatgcagctcc
	Tag	ctgtcgtggctctctccgcgag
	1NL+Tag	ctgtcgtggctctctccgcgaggttaatttgcatagtcac
	R48+Tag	ctgtcgtggctctctccgcgagctctcacacttctattatagag
	Left side donor	cctctgacacatgcagctcccgg
	Right side donor	ccgactggaaagcgggcagtg
	Right end fwd	tgtctctcactcggaagctagcgacatacc
	Left end rev	ggcgcttgacacctgcgtatgc
	LE18 rev	gcgtagcggagggatggtc
	Cam loop 1	ggtgcccttaaacgcctggatcc
	F1	cgatgccattgggatatatcaacgg
	2Tag	ctgtcgtggctctctccggagg
	2RE+Tag	ctgtcgtggctctctccggaggctctcacacttctattatagag
	2LE+Tag	ctgtcgtggctctctccggaggggtagtgatcttatttcattatg
	MM for Tag	acgcccggtagtgatcttatttcattatggtg
pHelR(mm)-Cam, pHelR(mm)-LacZ heteroduplex generation	Complement 1	/Bio/ggaagtggtaccagggcttctctagaataacagggacaccgcgctga
	C-A MM	/P/tcagcgcggtgtccctgttatcctagagaagccctggtaccacttcc
pHelR(mm)-GFP(-)-Puro heteroduplex generation	Complement 2	/Bio/ggaccatggtggcccgcggtaccgtcgactgcaggctga
	ACG MM	/P/tcagcctgcagtcgacggtaccgcgggccaccacggtcc
pHelR(mm)-GFP(+)-Puro heteroduplex generation	Complement 3	/Bio/ggaccgtggtggcccgcggtaccgtcgactgcaggctga
	ATG MM	/P/tcagcctgcagtcgacggtaccgcgggccaccatggtcc
DpnI digestion	DAM (+)	tcagcg/m6A/tctctccctgttaatctag/m6A/tcacatttggtaccag/m6A/atc c
	DAM (-)	gg/m6A/tctggtaccaaatgtg/m6A/tctagattaacagggagag/m6A/ /atcgctga
	Unmethylated (+)	tcagcgatctctccctgttaatctagatcacatttggtaccagatcc
	Unmethylated (-)	ggatctggtaccaaatgtgatctagattaacagggagagatcgctga
In vitro cleavage reactions	(+) Junction	tgtgcacgaatttcgtgcaccgggccactagtcctatataataaaagagaa/Fam /
	(-) Junction	/Fam/ttctcttttattatataggactagtggcccggtgcacgaaattcgtgcaca
	(+) Marker	tcctatataataaaagagaa/Fam/
	(-) Marker	/Fam/ttctcttttattatatagga

Supplementary Table 1. List of PCR primers and oligonucleotides

/P/: 5’Phosphate
/Bio/: 5’Biotin
/m6A/:N6-methyladenosine
/Fam/ : 6-Fam

Supplementary Note 1

Constructs

To create pHelR-CMV-Puro transposon donor plasmid, 602 bp DNA fragment (synthesized by Integrated DNA technologies (IDT)) containing *Puro* coding sequence was cloned into the *Mlu*I/*Pst*I site of the pHelR-MEGFP-IRES-Puro vector (synthesized by Bio Basic). To generate pHelR-Cam transposon donor plasmid, 883 bp DNA fragment (synthesized by IDT) containing *Cam* resistance gene was cloned into the *BamH*I/*Xba*I site of the pHelR[1] plasmid, replacing thereby the *Puro* marker gene and generating pHelR-CM1 plasmid. In the next step *Nde*I/*Nco*I fragment of the pHelR-CM1 plasmid was replaced with the 884 bp *Nde*I/*Nco*I fragment (synthesized by IDT) containing the 54 bp region (mismatch region) flanked by the two Nt.BbvCI recognition sites (**Supplementary Fig. 3**) creating thereby pHelR-Cam transposon donor. The mismatch region of the pHelR-Cam donor, used in heteroduplex plasmid generation, is located immediately downstream of the *Helraiser* LTS. It contains *Kpn*I and *Xba*I restriction enzyme recognition sites, the latter overlapping the mismatch position in heteroduplex donors. To generate pHelR-Cam-LacZ transposon donor plasmid 2578, bp PCR amplified fragment of pDS123 plasmid (gift of Dhruba Chattoraj) containing *R6K* bacterial origin of replication was cloned into the *Spe*I/*Nde*I site of the pHelR-CM1 plasmid, creating thereby pHelR-Cam-R6K vector. Next, 1385 bp DNA fragment (synthesized by IDT) containing pBR322 origin of replication followed by the above described mismatch region was cloned into the *Xba*I/*Nco*I site of pHelR-Cam-R6K plasmid to generate pHelR-Cam-ori vector. Finally, the 976 bp DNA fragment (synthesized by IDT)

containing LacZα fragment of β-galactosidase gene was cloned into the *BamH*I/*Pci*I site pHelR-Cam-ori plasmid to generate pHelR-Cam-LacZ donor plasmid. To create pHelR-GFP-Puro donor plasmid in which a CMV-GFP-IRES-Puro cassette was inserted between the *Helraiser* LTS and RTS, 3275 bp *Sph*I/*EcoR*I fragment (synthesized by Bio Basic) was inserted into the pUC57 plasmid backbone, creating thereby pUC57-GFP-Puro plasmid. The synthesized fragment contained *Helraiser* LTS and RTS flanking the CMV-eGFP-puro-IRES-β-globin terminator cassette. The internal ribosomal entry site (IRES) facilitates simultaneous expression of both the *GFP* and the *Puro* resistance genes from the same *CMV* promoter. eGFP sequence in this cassette was modified by changing the GFP start codon from methionine to glycine and by adding four amino acids downstream of the new start codon. The Kozak sequence was incorporated at the new eGFP start codon and the DNA sequence coding for additional amino acids contained *Nt.BbvC*I recognition site. Second *Nt.BbvC*I recognition site was placed 32 bp upstream. 46 bp region flanked by the two *Nt.BbvC*I sites constituted the mismatch region (**Supplementary Fig. 3**) used to generate heteroduplex donor plasmids. The mismatch region included *Pst*I and *Nco*I recognition sites, the latter overlapping the mismatch position in heteroduplex donors. Finally, the backbone of the pUC57-GFP-Puro was replaced by the 1588 bp *Mfe*I/*Sph*I DNA fragment (synthesized by IDT) containing chloramphenicol resistance gene and pBR22 origin of replication, generating thereby pHelR-GFP-Puro donor plasmid. To generate pHelR-MutGFP-Puro transposon donor, the start codon (atg) of the modified eGFP coding sequence in pHelR-GFP-Puro plasmid was changed to acg by replacing 686 bp of the pHelR-GFP-Puro plasmid with *Xho*I/*Age*I DNA fragment (synthesized by IDT) containing the point mutation. In addition to changing the start codon of modified eGFP gene from methionine to threonine, the introduced mutation abolished the *Nco*I

restriction site in the mismatch region of the pHelR-MutGFP-Puro plasmid (**Supplementary Fig. 3**). To generate pLexNHH control plasmid, the *Helraiser* transposase gene from the pHelR plasmid was cloned into the *Age*I/*Xho*I sites of the pLex plasmid (gift of Martin Gellert). The cloning procedure generated a frameshift at the beginning of the transposase coding sequence, abolishing thereby the expression of the *Helraiser* transposase from this plasmid.

Supplementary Note 2

Heteroduplex plasmid generation

Following the nicking reactions (~100 µg DNA), the gapped donor plasmids were generated by removing the nicked strand. This was done by incubation of the nicked strand with its biotinylated complement followed by the removal of the hybrid with streptavidin-magnetic beads (Roche). The gapped donors were then purified by phenol/chlorophorm extraction and EtOH precipitation. The efficiency of making gapped DNA plasmids was monitored by the loss and restoration of the restriction enzyme cleavage site between the two nicking sites (*Kpn*I for pHelR-Cam and pHelR-Cam-LacZ, and *Pst*I for pHelR-GFP-Puro) (**Supplementary Fig. 1**). In the next step a 100-fold excess of the phosphorylated oligo containing the mismatched nucleotide was used in the annealing reaction with the gapped donor plasmids, followed by the overnight incubation with T4 DNA ligase (NEB) at 16°C. Where it was possible (pHelR(mm)-Cam and pHelR(mm)-Cam-LacZ), to remove possible leftovers of the unmodified template plasmids, the ligation reactions were digested with the single-cutting enzyme (*Xba*I), whose recognition site overlapped the mismatch position. In this manner, the heteroduplex transposon donors that were resistant to the enzymatic cleavage due to the change in enzyme recognition sequence were preserved,

whereas the original template plasmids were linearized. The products containing nicks and gaps, as well as linearized template plasmids, were then removed from the reaction by Exonuclease III (NEB) digestion (700U) for 2 hours at 37°C. Next, the reaction products were run on 1% agarose gel stained with Midori Green (Nippon Genetics) and the DNA band containing heteroduplex donor plasmids was excised from the agarose gel. Heteroduplex donor plasmids were then isolated from the agarose gel using Nucleospin Gel and PCR clean-up kit (Macherey-Nagel). The efficiency of making heteroduplex donors was monitored by the resistance to the digestion with the restriction enzymes whose recognition sites overlapped the mismatch position (*Xba*I in the case of pHelR-Cam and pHelR-Cam-LacZ, and *Nco*I in case of pHelR-GFP-Puro). In the case of pHelR(mm)-GFP(+)-Puro, where restriction enzyme cleavage site did not overlap the mismatch position, the quality of the generated heteroduplex plasmid donors was verified by the sequencing of the exchanged strand.

Supplementary Note 3

Single-stranded plasmid generation

One strand of the supercoiled plasmid DNA (~40 µg DNA) was nicked in an overnight reaction (Nt.BspQI (NEB) was used to nick the plus and Nb.BsmI (NEB) was used to nick the minus strand), followed by the heat inactivation of the enzyme according to the manufacturer's protocol. In the next step, Exonuclease III (NEB) was added (400U) to the nicking reaction and incubated for 2 hours at 37°C in order to degrade the nicked strand. Exonuclease III digestion reactions were then run on a 1% agarose gel stained with 2 µg /ml EtBr and visualized under the blue light. The prominent DNA band containing ssDNA was excised and ssDNA was extracted from the agarose

gel slice with the Nucleospin gel extraction kit (Macherey-Nagel inc.). The efficiency of each step in this procedure (nicking reaction, Exonuclease III digestion and ssDNA extraction from the agarose gel) was monitored by agarose electrophoresis. The quality of the isolated ssDNA was tested in S1 nuclease and Exonuclease I assay. S1 nuclease digestion completely degraded assayed ssDNA, while it remained unaffected by the Exonuclease I digestion. This confirmed that the majority of the isolated ssDNA was in covalently closed form.

Supplementary Note 4

PCR

Unless stated otherwise, 200 ng of the isolated LMW DNA and 200 nm primers (each) were used per 50 µl PCR reaction. PCR primer sequences are listed in the **Supplementary Table 1**.

Circle detection PCR. PCR reactions (**Fig. 2c** and **Supplementary Fig. 4**) with primers Hel1 and Hel5 were performed as described previously[1], with modifications. Temperature profile was 95°C 2 min followed by 45 cycles of (95°C 20 s, 57°C 15 s, 72°C 10 s). The final elongation was performed at 72°C for 3 min. When detecting ss transposon donors (**Fig. 2b**) 20 PCR cycles were performed.

Donor detection PCR. PCR reactions (**Fig. 2a**) were performed using Hel1 and pUC57 primers. Temperature profile was 95°C 2 min followed by 20 cycles of (95°C 20 s, 57°C 15 s, 72°C 10 s). The final elongation was performed at 72°C for 3 min.

Strand-specific PCRs. PCR (**Fig. 2d** and **e**) and primer design were according to previously described method[2] with some modifications. First, 15 cycles of asymmetric PCR were performed using only one 2 nM primer per 50 µl PCR reaction; The used primer hybridized specifically either

with the plus or the minus strand of the transposon donor plasmids or *Helraiser* circles. In addition, the primers carried a 21 bp 5' overhag (Tag) which was not specific to any of the template DNA used in the PCR reactions. The temperature profile was 95°C 2 min followed by 15 cycles of (95°C 20 s, 50°C 15 s, 72°C 40 s). The final elongation was performed at 72°C for 5 min. In the second round 2.5 µl of the first-round PCR was used and two 200 nM primers (each). One of the second-round primers was hybridizing specifically with the transposon donor plasmids or the *Helraiser* circles, while the other primer was specific to the Tag overhang present only on the amplification products generated in the first asymmetric PCR, but not on the transposon donors or circles. Melting temperatures of the primers from the first round (~49°C) were significantly lower than the melting temperatures of the primers used in the second round (~63°C) of PCR. Therefore, in addition to their low concentration, the primers used in the first round were not expected to produce amplification products using the temperature profile of the second PCR. To exclusively detect strand-specific transposon-end junctions, the same principle described previously for the circle detection PCR[1] was applied to the first and the second-round primer design. The temperature profile of the second round PCR was: 95°C 2 min followed by 30 cycles of (95°C 20 s, 62°C 15 s, 72°C 15 s). The final elongation was performed at 72°C for 5 min. Primer set used for the SS-TJD PCR (**Fig. 2d**) was 1NL+Tag (binding to the plus strand) or R48+Tag (binding to the minus strand) in the first round of PCR. In the second round of PCR, Tag primer was used in combination with Right end fwd primer (where 1NL+Tag was used in the first PCR) or with Left end rev primer (where R48+Tag was used in the first PCR). Primer set used for the SS-PCR (**Fig. 2c**) was 1NL+Tag or R48+Tag in the first round of PCR. In the second round of PCR, Tag primer was used in combination with Left side donor primer (where 1NL+Tag was used in the first PCR)

or with Right side donor primer (where R48 + Tag was used in the first PCR). Prior to performing the strand-specific PCR assays, the efficiency and strand specific amplification of this method was evaluated using complementary 200 nt long oligonucleotides (synthesized by IDT) containing primer binding sites from transposon donor plasmids (data not shown). Sequencing of multiple plus and minus strand specific TJD-PCR products performed with the LMW DNA from transfections with ds and heteroduplex donors, revealed that they contained predominantly precise transposon junctions (data not shown).

Supplementary Note 5

DpnI replication assay

Following the *Dpn*I digestion (there are 9 *Dpn*I restriction sites on transposon donor plasmids) and electroporation of the isolated LMW DNA, electroporated cells were plated on agar plates containing 25 µg/ml Cloramphenicol (*Cam*), 40 µg/ml X-gal and 0.5mM IPTG. *Helraiser* circles recovered from white *Cam* resistant colonies were analyzed by DNA sequencing. Since it was demonstrated previously that *dam*-methylation status of non-replicating plasmids in mammalian cells is maintained for at least six months[3], and our plasmids did not contain any sequences that could serve as replication origins in HEK293T cells, it is not likely that the loss of dam-methylation on *Helraiser* circles occurred in transposase-indipendent manner. To confirm the lack of methylation, the products of the *Dpn*I reactions were digested with *Mbo*I, resulting in the complete loss of white colonies as expected (**Supplementary Fig. 2**)). When *Mbo*I (NEB) digestion was performed, 5U of *Mbo*I was added to the *Dpn*I digestion and incubated for 3 hours 63°C prior to electroporation to *E. coli*. Efficiency of the *Dpn*I digestion of the *Dam* methylated,

unmethylated and hemimethylated DNA was evaluated using the double-stranded 47 bp oligonucleotides (**Supplementary Table 1**) that contained three fully methylated, hemimethylated or unmethylated sites. 10 pmol of the double-stranded oligo was digested overnight at 37 °C with 10U of *Dpn*I or with 5U of *Mbo*I enzyme in the final volume of 13 µl. The digestion products were resolved on Novex 10% TBE gel (Thermo Fisher Scientific). To test if the *Dam* methylated ssDNA plasmids can be digested with the *Dpn*I enzyme, 300 ng of the single-stranded pHelR-Cam plasmid was digested for 4 hours with 10U of the *Dpn*I enzyme. Visualization of the reaction products on an agarose gel revealed that both, ss and ds plasmids, were digested by *Dpn*I (data not shown). To test if the *Dam* methylated and *Dpn*I digested ssDNA plasmids can give rise to *E. coli* colonies after the electroporation, we digested 10 ng of the single-stranded pHelR-Cam plasmid with 5U of *Dpn*I for for 4 hrs in the final volume of 30 µl. 1 µl of the *DpnI* digestion was electroporated to *E. coli*, but no Cam resistant colonies were formed. In contrast, electroporation of the same quantity of the undigested ss pHelR-Cam to *E. coli* yielded about 100-300 colonies per plate, whereas electroporation of the ds form of the same plasmid yielded about 2000 colonies. To investigate *Dpn*I digestion of the ssDNA in conditions similar to the ones used in the assay with isolated LMW DNA, 1ng of the *Dam* methylated single-stranded pHelR-Cam plasmid was mixed with the *Dam* methylated pFHelR helper plasmid (500 ng) and the pHelR-Cam (500 ng) and digested overnight with 10U of the *Dpn*I enzyme. The subsequent electroporation of the digestion reaction to *E. coli* did not yield any colonies.

Supplementary Note 6

***In vitro* formation and PCR detection of transposon end junctions**

Following the Proteinase K digestion, in vitro junction-formation reactions were cleaned-up with QIAquick PCR purification kit (Qiagen) and purified DNA was used in PCR. To detect transposon end junction formation (**Fig. 5b and c**) nested CD-PCR was performed. In the first round, 200 nM primers (each) Hel1 and Cam loop 1 were used in the total volume of 50 µl. The temperature profile for the nested PCR was: 95°C 2 min followed by 30 cycles of (95°C 20 s, 62°C 15 s, 72°C 20 s). The final elongation was performed at 72°C for 3 min. The second, PCR was performed with 200 nM nested primers (each) Hel5 and LE18 rev in the total volume of 50 µl. 1 µl of the 1:100 dilution of the first PCR was used per reaction. The temperature profile for the nested PCR was: 95°C 2 min followed by 26 cycles of (95°C 20 s, 56°C 15 s, 72°C 10 s). The final elongation was performed at 72°C for 3 min. SS-TJD PCR (**Fig. 5e**) was performed in three rounds of PCR. The first round asymmetric PCR was performed in 15 cycles using 2 nM primers (2LE+Tag (binding to the plus strand) or 2RE+Tag (binding to the minus strand)) in a 50 µl reaction. The temperature profile of the asymmetric PCR was as described above. The second round of the strand-specific PCR was performed in 25 cycles using the primer combinations Cam loop 1 and 2Tag where 2LE+Tag primer was used in the first asymmetric PCR and MM for Tag and 2Tag where 2RE+Tag was used in the first asymmetric PCR. The PCR conditions and the temperature profile for the second round of PCR were as described above. In the third round, nested PCR was performed with 200 nM primers (each) Hel5 and LE18 rev in the total volume of 50 µl. 1 µl of the 1:50 dilution of the second PCR was used per reaction. The temperature profile for the nested PCR was: 95°C 2 min followed by 28 cycles of (95°C 20 s, 56°C 15 s, 72°C 10 s). The final elongation was performed at

72°C for 3 min. PCR products were visualized on an agarose gel. Transposon end junction formation was confirmed by DNA sequencing.

Transposon end junctions generated from the heteroduplex donor were detected with CD-PCR (**Fig. 5f**) using 200 nM primers (each) F1 and Hel5 in the total volume of 50 µl. The temperature profile for the PCR was: 95°C 2 min followed by 45 cycles of (95°C 20 s, 55°C 15 s, 72°C 15 s). The final elongation was performed at 72°C for 5 min. The obtained PCR product of the expected size was isolated from the agarose gel, cloned into the pTZ57R/T cloning vector (Thermo Fisher Scientific) and electroporated into electrocompetent ElectroMAX DH10B *E. coli* cells (Thermo Fisher Scientific). Plasmids containing cloned PCR fragments were isolated from the individual *E.coli* colonies and sequenced.

Supplementary References

1. Grabundzija I*, et al.* A Helitron transposon reconstructed from bats reveals a novel mechanism of genome shuffling in eukaryotes. *Nat Commun* **7**, 10716 (2016).

2. Zhong S*, et al.* Frequent detection of the replicative form of TT virus DNA in peripheral blood mononuclear cells and bone marrow cells in cancer patients. *J Med Virol* **66**, 428-434 (2002).

3. Coelho-Castelo AA*, et al.* Tissue distribution of a plasmid DNA encoding Hsp65 gene is dependent on the dose administered through intramuscular delivery. *Genet Vaccines Ther* **4**, 1 (2006).
